# Role of the RBMS Family in Different Cancers and Research Progress

**DOI:** 10.7150/ijms.118386

**Published:** 2026-01-23

**Authors:** Hanchi Wu, Rui Hou, Yuhan Zhang, Hanfang Fan, Junying Xu, Huiyu Wang

**Affiliations:** 1 The Affiliated Wuxi People's Hospital of Nanjing Medical University, Wuxi People's Hospital, Wuxi Medical Center, Nanjing Medical University, Wuxi 214023, China.; 2 Corresponding author: Huiyu Wang, Department of Oncology, The Affiliated Wuxi People's Hospital of Nanjing Medical University, Wuxi People's Hospital, Wuxi Medical Center, Nanjing Medical University, 299 Qingyang Road, Wuxi 214023, China.

**Keywords:** RNA-binding protein, cancer, tumor microenvironment

## Abstract

RNA-binding proteins (RBPs), as posttranscriptional regulators, can modulate the activity and stability of target RNAs and participate in the whole life cycle of RNA processing, localization, modification and translation. RNA-binding motif single-stranded interacting proteins (RBMSs) comprise a critical subgroup within the RNA-binding protein (RBP) family, sharing the same domain characteristics as other RBPs. Several studies have shown that RBMSs can participate in tumorigenesis and tumor progression through mechanisms such as regulating the expression of oncogenes, growth factors and cell cycle proteins. In this paper, we reviewed the role of RBPs and related research progress in breast, prostate, lung, liver, gastric and colorectal cancers.

## Introduction

RBPs are a class of critical intracellular regulators that play widespread roles in post-transcriptional gene regulation, including RNA splicing, transport, subcellular localization, and translation. Based on their functional role, RBPs are categorized into several conserved families, such as Hu-antigen R (HuR), heterogeneous nuclear ribonucleoproteins (hnRNPs), serine/arginine-rich splicing factors (SRSFs), and RNA-binding motif single-stranded interacting proteins (RBMSs)^1^. Notably, RBMSs are involved in several aspects of RNA metabolism and carry out their functions by binding to single-stranded DNA/RNA and regulating pretranscription at the gene level. Mammalian RBMSs comprise three members: RBMS1, RBMS2, and RBMS3. These proteins bind to specific nucleic acid sequences and regulate critical biological processes including DNA replication, transcriptional regulation, cell cycle progression, and apoptosis^2^. Growing evidence highlights the significant impact of RBMSs on malignant tumor progression. They contribute to cancer metastasis, shape the immune microenvironment, modulate gene expression profiles, and affect clinical prognosis (Table 1). For example, RBMS1 modulates hepatocellular carcinoma development through the ferroptosis pathway; RBMS2 enhances chemosensitivity in breast cancer by stabilizing tumor suppressor gene mRNAs; and RBMS3 inhibits epithelial-mesenchymal transition (EMT) via suppression of the Wnt signaling pathway. In this paper, we review the biological functions of RBMS family members in different tumors with the aim of providing new ideas for further exploration of the mechanism of malignant tumor development and identifying new therapeutic targets.

## 1. RBMS1

### 1.1 RBMS1 and liver cancer

**Figure 1:**
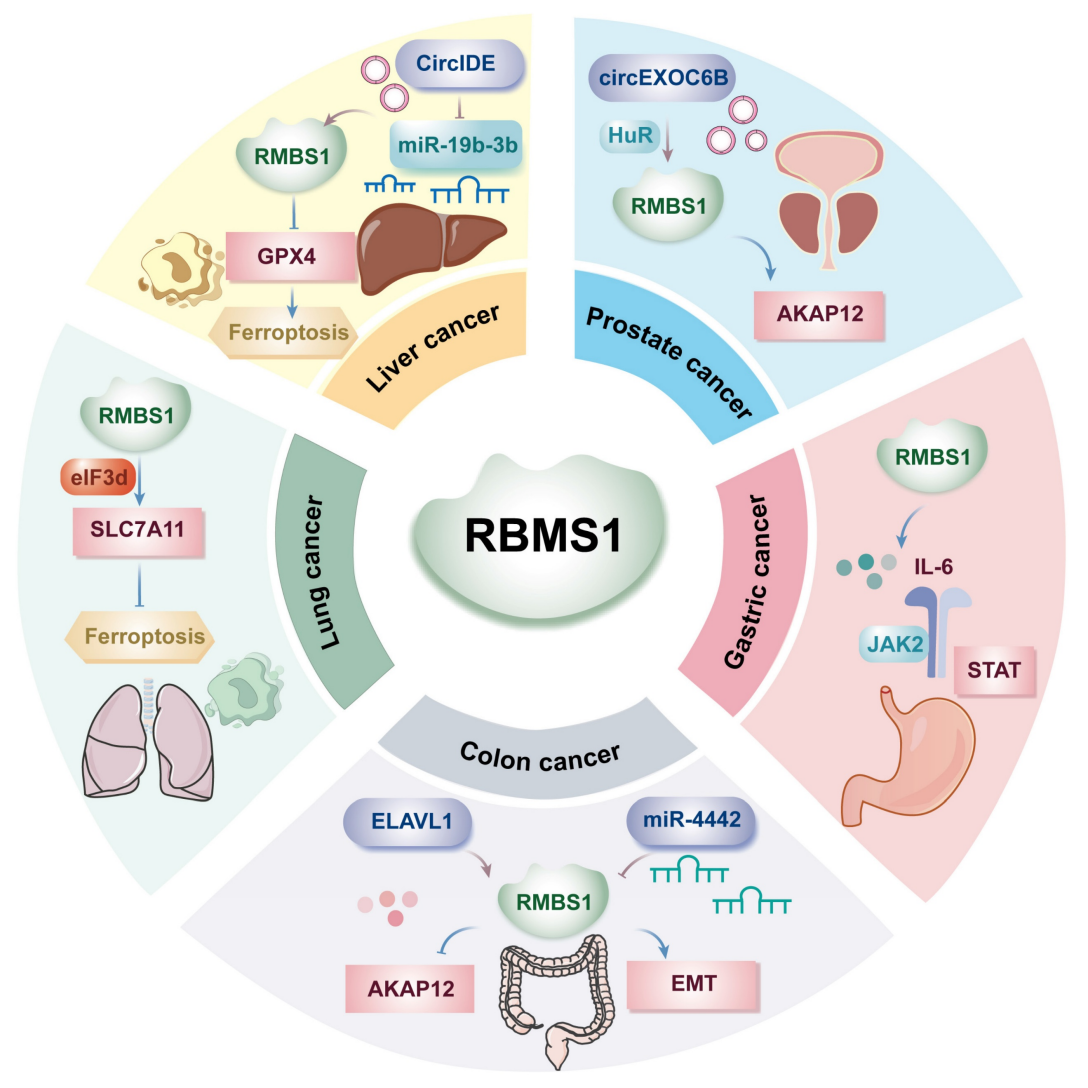
Roles of RBMS1 in different cancer.

Ferroptosis is an iron dependent, novel mode of programmed cell death distinguished from apoptosis, cell necrosis, and cell autophagy and is closely related to the development of many malignant tumors. Hepatocellular carcinoma is the fourth most common malignant tumor in China, and its mortality rate is the second highest among malignant tumors. Several studies have shown that RBMS1 is involved in the development of hepatocellular carcinoma as a regulator of iron-mediated death (ferroptosis). ZHAI *et al.* found by western blotting that RBMS1 regulates the stability of glutathione peroxidase (GPX4) mRNA and reduces the level of GPX4 through its 3'-UTR, which promotes ferroptosis and inhibits the proliferation of hepatocellular carcinoma cells^3^ (Figure 1). Clinically, patients with high circIDE expression had better overall survival and recurrence-free survival, and downregulation of RBMS1 was associated with worse survival in hepatocellular carcinoma patients. The team further explored the underlying mechanism and revealed that the functions of RBMS1 in promoting ferroptosis and inhibiting tumor growth in hepatocellular carcinoma were regulated by the circIDE/miR-19b-3p axis. circIDE upregulates RBMS1 in hepatocellular carcinoma by sponging miR-19b-3p, which decreases the expression of GPX4, inhibits its ability to reduce lipid peroxidation, and promotes ferroptosis^3^. The RBMS1/circIDE/miR-19b-3p axis, as a regulator of ferroptosis, may be a potential therapeutic target in hepatocellular carcinoma.

### 1.2 RBMS1 and gastric cancer

Gastric cancer ranks as the second most common cause of cancer-related death globally, with incidence and mortality rates trailing only lung cancer in China. The metastasis of gastric cancer is a major factor leading to death, and exploring the molecular mechanism of gastric cancer metastasis is highly important for the early diagnosis, treatment, and prognosis evaluation of gastric cancer. Liu *et al.* demonstrated the involvement of the immune microenvironment in gastric cancer metastasis, highlighting the highly activated IL-6/JAK2/STAT pathway during malignant tumor metastasis (Figure 1). Additionally, upregulated RBMS1 expression in gastric cancer tissues and cells correlates positively with lymph node metastasis at T3/T4 stages and recurrence risk. Upon further exploration of the molecular mechanism of RBMS1 in regulating gastric cancer metastasis, IL-6 was found to be significantly upregulated in the culture supernatant of gastric cancer cells overexpressing RBMS1, suggesting that RBMS1 promotes the migration and proliferation of gastric cancer cells by regulating autocrine IL-6 signaling. Moreover, RBMS1 and IL-6 levels were positively correlated, and inhibition of RBMS1 significantly decreased the level of IL-6; in addition, the downstream target of IL-6, JAK2/STAT, was downregulated^4^. In addition, after binding to MYC, RBMS1 affected the transactivation of IL-6 and the activation of the IL-6/JAK2/STAT downstream signaling pathway by affecting histone modifications in the promoter region, promoting the migration and invasion of gastric cancer cells. The above study suggested that RBMS1 is an important biomarker for gastric cancer metastasis and that its high expression is associated with a poor prognosis.

In addition, Yue *et al.* showed that the KCNQ1OT1/miR-378a-3p/RBMS1 axis is a novel prognostic biomarker for immune cell infiltration in gastric cancer. Long non-coding RNAs (lncRNAs), acting as miRNA “sponges,” are termed competitive endogenous RNAs (ceRNAs) and can reduce the inhibitory effect of miRNAs on target mRNAs^5^. By analyzing public databases, YUE *et al.* explored a prognostic mRNA-miRNA-lncRNA network and revealed that lncRNA KCNQ1OT1 acts as a ceRNA by competitively binding to the tumor suppressor miR-378a-3p via the KCNQ1OT1/miR-378a-3p/RBMS1 axis, resulting in upregulated RBMS1 expression in tumors^6^. These findings suggest that lncRNA KCNQ1OT1 may serve as a ceRNA that promotes RBMS1 expression through sequestration of miR-378a-3p. Furthermore, the study assessed the relationships among miR-378a-3p, lncRNA KCNQ1OT1, and immune infiltration in gastric cancer. During immune cell infiltration, the expression of miR-378a-3p was inversely correlated with the expression of RBMS1. RBMS1 expression was positively correlated with the immune response, miR-378a-3p was negatively correlated with the immune response, and lncRNA KCNQ1OT1 expression was also negatively correlated with the infiltration level of cells such as NK CD56dim cells. According to recent evidence, molecularly targeted therapies promote NK cell-mediated tumor cell killing^7^. The findings from Yue *et al.* suggested that RBMS1 may suppress gastric cancer progression by enhancing the anti-tumor immune response, and that the lncRNA KCNQ1OT1/miR-378a-3p/RBMS1 axis is an important prognostic factor in gastric cancer and has the potential to be a therapeutic target. However, a larger sample size is needed to validate this phenomenon in gastric cancer tissues.

In summary, studies by Liu *et al.* demonstrated that RBMS1 promotes metastasis in advanced-stage (T3/T4) gastric cancer through activation of the IL-6/JAK2/STAT3 signaling pathway. In contrast, Yue *et al.* reported that the KCNQ1OT1/miR-378a-3p/RBMS1 axis may improve patient prognosis by enhancing immune cell infiltration. The apparent dual role of RBMS1 in tumor progression remains unclear and may reflect differences in immune microenvironment composition and/or tumor stage. Future studies employing transcriptomic profiling to compare tumors with high versus low RBMS1 expression are warranted to elucidate the microenvironment-specific regulatory networks governed by RBMS1.

### 1.3 RBMS1 and colon cancer

Colon cancer is a malignant tumor of the digestive system, and the latest statistics show that colorectal cancer is the third most common malignant tumor and the fourth leading cause of cancer-related death worldwide. It is also the fourth most common cancer in men and the third most common cancer in women worldwide^8^. The role of RBMS1 in colon cancer metastasis has been investigated, and clinical data suggest that low RBMS1 expression is associated with a poor clinical prognosis, while RBMS1-mediated regulation of mRNA stability is decreased in highly metastatic colon cancer^9^. Genome-wide RNA stability measurements in control and RBMS1 knockdown cells revealed a similar reduction in the stability of putative RBMS1 regulators upon RBMS1 silencing, identifying RBMS1 as a posttranscriptional regulator of RNA stability with clear implications for colorectal cancer progression.

RBMS1 is highly expressed in the SW480 colon cancer cell line, which has a significantly reduced hepatic colonization capacity compared to other colon cancer cell lines. YUE *et al.* irCLIPed endogenous RBMS1 in SW480 cells and identified hundreds of high-confidence RBMS1 binding sites (3' UTRs) in the transcriptome, which is consistent with the role of RBMS1 as a posttranscriptional regulator of RNA stability and suggests that RBMS1 3' UTR binding may lead to increased RNA stability^10^. Further analysis revealed that 72% of RBMS1-bound 3' UTRs *in vivo* were also bound by ELAV-like RNA-binding protein 1 (ELAVL1) and that knockdown of ELAVL1 resulted in significant downregulation of RBMS1 targets, suggesting that direct binding of RBMS1 to 3' UTRs in mRNAs is accompanied by interactions with other stabilizers, such as ELAVL1, that together maintain mRNA stability. In contrast, RBMS1 expression was almost completely silenced in the highly liver-metastatic cell line LS174T, which has approximately 100-fold greater metastatic capacity than the SW480 cell line. Knockdown of RBMS1 expression in cells endogenously expressing RBMS1 resulted in increased hepatic colonization. In addition, epithelial-mesenchymal transition (EMT) signature genes were also expressed at relatively low levels in the LS174T cell line.

YU *et al.* showed that A-kinase anchor protein 12 (AKAP12) and Syndecan binding protein (SDCBP) are considered downstream targets of RBMS1, with AKAP12 being more significantly downregulated in highly metastatic colorectal cancers or RBMS1 knockdown cell lines^10^. Silencing of RBMS1 in SW480 cells resulted in downregulation and decreased stability of AKAP12 and SDCBP mRNA. In contrast, overexpression of RBMS1 in LS174T cells resulted in upregulation and elevated stability of these targets. However, further silencing of AKAP12 failed to result in increased metastasis in RBMS1 knockdown cells, whereas AKAP12 expression was already downregulated in RBMS1 knockdown cells^9^. qRT‒PCR was performed to validate these results, and a significant decrease in AKAP12 expression with disease progression was observed. Consistent with the finding that RBMS1 is a regulator of AKAP12, a highly positive and significant correlation was observed between the expression of these two genes. These findings suggest that AKAP12 acts as a metastasis suppressor downstream of RBMS1 and is associated with the development of malignant metastasis (Figure 1). This study further explored the mechanism of RBMS1 silencing in highly metastatic LS174T colon cancer cells and revealed that RBMS1 expression was closely associated with promoter acetylation; deacetylase was most commonly upregulated in highly metastatic cells; and the use of the deacetylase inhibitor trichostatin A (TSA) increased RBMS1 expression, indicating that deacetylase-mediated transcriptional repression may be a possible mechanism leading to RBMS1 silencing.

It has been shown that miR-4442 may affect colorectal cancer metastasis and prognosis by regulating RBMS1^11^. In Shibamoto's study, RBMS1 was shown to regulate the EMT and metastatic ability of colon cancer cells (Figure 1). miR-4442 expression in cancer tissues was negatively correlated with RBMS1 mRNA expression in cancer tissues, suggesting that RBMS1 mRNA is a direct target gene of miR-4442. Overexpression of miR-4442 decreased the levels of RBMS1 and E-cadherin, which are inhibitors of EMT in colon cancer, suggesting that miR-4442 regulates epithelial mesenchymal transition in colon cancer through RBMS1. In summary, miR-4442 increases the malignant potential of colon cancer, and miR-4442 inhibitors may be potential targets for colon cancer treatment to improve patient prognosis.

### 1.4 RBMS1 and lung cancer

Ferroptosis, an iron-dependent form of non-apoptotic cell death induced by membrane damage controlled by cystine depletion and massive lipid peroxidation, plays a key role in tumor suppression^12^. Previous studies have shown that the expression of RBMS1 varies in different malignant tumor tissues; for example, RBMS1 is expressed at low levels in colorectal cancer tissues, while the level of RBMS1 in lung cancer tissues is significantly greater than that in adjacent normal tissues. Zhang *et al.* revealed that RBMS1 may exert a protumorigenic effect on lung cancer cells by inhibiting ferroptosis. Their results showed that RBMS1 established a bridge between the 3'UTR and 5'UTR of solute carrier family 7 member 11 (SLC7A11) by directly interacting with the translation initiation factor eIF3d, thereby promoting the translation of SLC7A11, increasing the intracellular glutathione content, and the glutathione peroxidase GPX4 utilizes glutathione to reduce lipid peroxidation and thereby inhibit ferroptosis^13^ (Figure 1). Zhang *et al.* suggested that RBMS1 modulates the level of ferroptosis in lung cancer cells not by regulating the stability of mRNAs but also by regulating the translation of SLC7A11, which in turn affects the biological behavior of tumors.

The context-dependent role of RBMS1 across cancers is noteworthy. Unlike its function as a tumor suppressor in colon cancer—where it stabilizes mRNAs of metastasis suppressors like AKAP12—RBMS1 exhibits oncogenic properties in lung cancer by promoting the translation of SLC7A11^9^, ^13^. This functional dichotomy underscores that the biological outcome of RBMS1 is not intrinsic but is determined by its tissue-specific interaction partners (e.g., eIF3d in lung cancer vs. ELAVL1 in colon cancer) and the pathways regulated by its downstream targets.

This study further explored the effects of RBMS1 modulators on ferroptosis and sensitivity to radiotherapy in lung cancer cells and revealed that nortriptyline hydrochloride reduced the level of RBMS1 in lung cancer cells, promoted ferroptosis, and resensitized radiation-resistant lung cancer cells to radiotherapy. These results provide compelling evidence for the therapeutic potential of RBMS1 inhibitors, particularly in combination with radiotherapy, to enhance tumor cell killing. This implies that RBMS1 not only regulates ferroptosis but may also serve as a novel therapeutic target by modulating radiotherapy sensitivity. Additionally, emerging evidence suggests that RBMS1 depletion can reduce the expression of programmed death-ligand 1 (PD-L1), thereby stimulating cytotoxic T cell-mediated antitumor immunity^14^. However, the clinical application of RBMS1 inhibitors remains in its early stages, and further experimental data and clinical trials are necessary to evaluate their efficacy and safety. The development of specific RBMS1 inhibitors represents an important future research direction. Their therapeutic effects will need to be assessed across different cancer types. It will also be essential to investigate combination strategies involving RBMS1 inhibition with other treatment modalities—such as immunotherapy and chemotherapy—to optimize therapeutic efficacy and improve clinical outcomes.

### 1.5 RBMS1 and prostate cancer

Prostate cancer is the most common malignant tumor in older men, and early detection of limited prostate cancer usually results in a better prognosis; however, for metastatic castration-resistant prostate cancer, the prognosis is poorer^15^. Bone metastasis is the most common site of metastasis in prostate cancer patients and is the cause of death in the majority of patients. Therefore, elucidating the mechanisms involved in prostate cancer development and metastasis is crucial for its treatment.

A study by Zhang *et al.* revealed that circEXOC6B, which originates from the EXOC6B gene, is expressed at low levels in prostate cancer tissues and that low circEXOC6B expression predicts advanced pT stage and a poor prognosis. Mechanistic studies have shown that circEXOC6B acts as a protein scaffold that binds to the typical RNA-binding proteins RBMS1 and HuR, regulates the stability and metabolism of its target mRNAs, and further enhances the expression of kinase-anchored protein 12 (AKAP12) to inhibit prostate cancer metastasis^15^ (Figure 1). A recent study revealed that a pair of short, inverted repeats on flanking introns partially promotes circularization of circEXOC6B, a novel mechanism by which circEXOC6B inhibits prostate cancer metastasis, providing new insights into the molecular process of circRNA generation and offering new potential targets for the diagnosis and treatment of PCa patients.

Previous studies by Dankert's team demonstrated that RBMS1 expression is downregulated in prostate cancer tissues compared to corresponding normal tissues, possibly because of direct regulation of endogenous RBMS1 expression in PCa cell lines by the oncogenic miRNA miR-106b^16^. In addition, this study identified, for the first time, the tumor-suppressor properties of RBMS1 in LNCaP and DU145 prostate cancer cells, namely, its ability to inhibit cell growth, promote wound healing, and increase colony formation ability; this study identified a novel role for RBMS1 in prostate cancer and performed deletion of RBMS1 to clarify the pathogenesis of PCa.

## 2. RBMS2

### 2.1 RBMS2 and breast cancer

**Figure 2:**
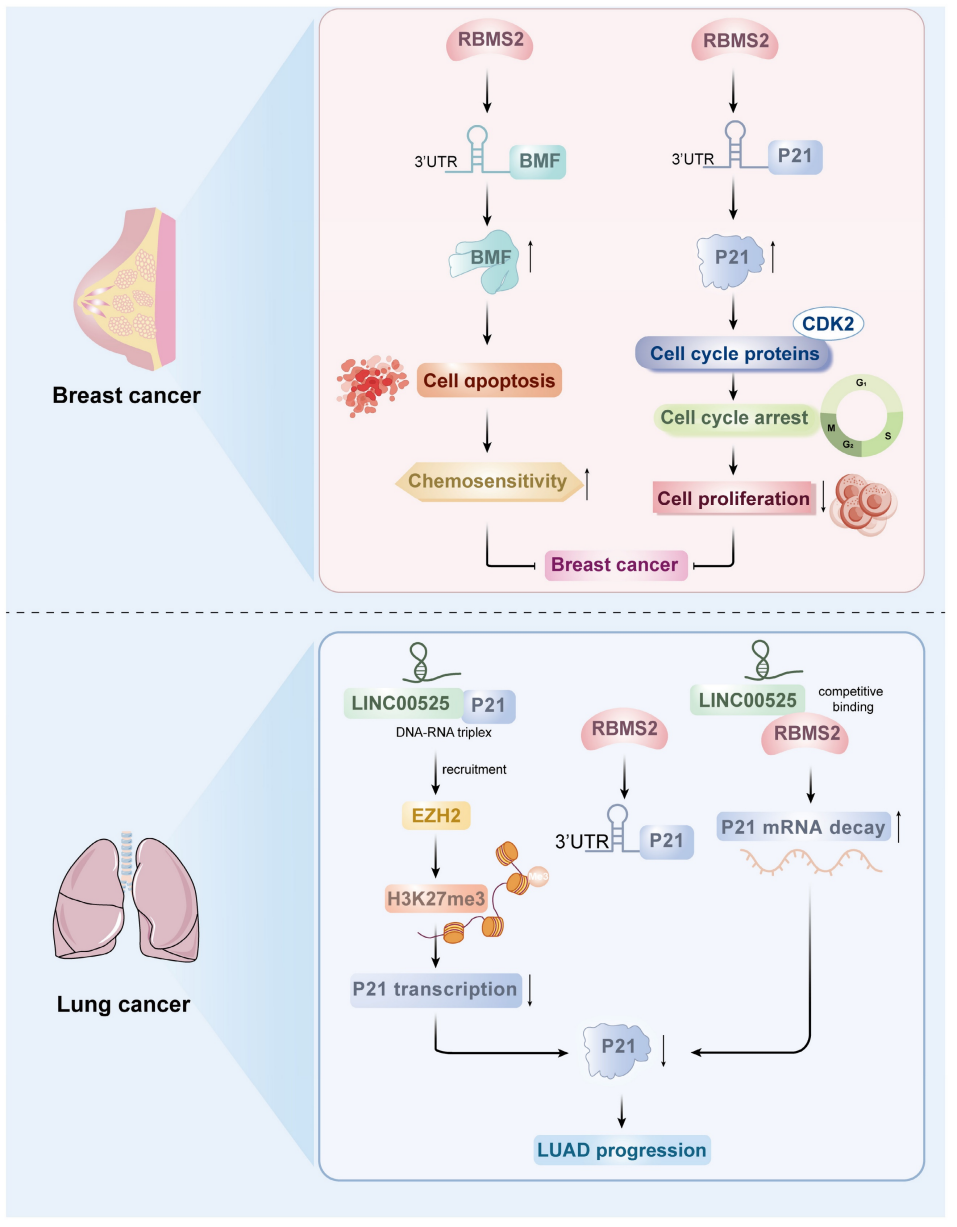
Roles of RBMS2 in breast cancer and lung cancer.

Several studies have shown that RBMS2 plays a role in cancer inhibition in a variety of malignant tumors^17^. According to a number of studies, RBMS2 expression is lower in breast cancer tissues than in normal tissues, suggesting that RBMS2 is a useful biomarker for breast cancer^18^. RBMS2 can improve the stability of BMF mRNA by binding to the AU-rich element of the 3'-UTR of Bcl-2 modifying factor (BMF), which is a proapoptotic factor that has been associated with a variety of cellular activities, including chemosensitivity. It was found that overexpression of RBMS2 increased the sensitivity of breast cancer cells to doxorubicin and induced apoptosis, while inhibition of RBMS2 expression had the opposite effect^17^. On the basis of the mRNA sequencing results, the team further found that BMF mRNA expression was significantly upregulated after RBMS2 was overexpressed in breast cancer cells. In addition, Xu *et al.* found that BMF expression was significantly downregulated in breast cancer tissues compared with paracancerous tissues and that BMF expression was significantly positively correlated with RBMS2 expression via analysis of the TCGA database, suggesting that BMF may be a potential target of RBMS2^17^. In the absence of doxorubicin, knocking down BMF alone promoted cell growth *in vitro* and *in vivo*; depletion of BMF may be one of the causes of resistance to doxorubicin. It is hypothesized that RBMS2 may enhance the sensitivity of breast cancer cells to doxorubicin by inducing apoptosis regulated by BMF expression. This study revealed that the expression of RBMS2 can increase the stability of BMF mRNA and enhance the responsiveness of tumor cells to doxorubicin, which provides a new potential target for the treatment of breast cancer (Figure 2).

The tumor suppressor protein P53-related pathway is an important pathway in the regulation of tumor proliferation. SUN *et al.* reported that the P53 pathway is the main pathway regulated by RBMS2, and the cell cycle inhibitory protein P21, an important component of the P53 pathway, is significantly upregulated after the overexpression of RBMS2. P21 inhibits cell proliferation function primarily through binding and inhibiting various cell cycle proteins (e.g., CDK2), which leads to growth arrest at specific stages of the cell cycle. In this study, the tumor suppressor RBMS2 was shown to act by stabilizing P21 mRNA in breast cancer. To explore the potential of RBMS2 to act as a tumor suppressor in breast cancer by inhibiting P21 expression, small interfering RNA or control genes were transfected into RBMS2-overexpressing SUM-1315 and MCF-7 cells, and the transfection efficiency was verified by qRT‒PCR and western blotting (Figure 2). According to the colony formation assays, the average number of colonies was reduced after RBMS2 overexpression. The number of cells was restored after interfering with the expression of P21. In addition, overexpression of RBMS2 in the MCF-7 and SUM-1315 cell lines significantly upregulated P21 expression, as determined by qRT‒PCR and western blotting. Similarly, P21 protein and mRNA levels were significantly decreased after RBMS2 was knocked down. Immunohistochemical staining also confirmed that P21 expression was positively correlated with RBMS2 expression. These findings suggest that P21 is an important target of RBMS2 that suppresses the proliferation of breast cancer cells, suggesting that P21 could be a new target for breast cancer treatment^18^.

### 2.2 RBMS2 and lung cancer

Lung cancer is the most common cause of cancer-related death worldwide, and lung adenocarcinoma is the most common histologic type^19^. LncRNAs play a crucial role in various cancers and can exert biological effects through interactions with DNA, RNA, and proteins, participating in the progression of many malignant tumors^20^-^22^. LINC00525 has been found to be upregulated in a variety of cancers, and its high expression is associated with high tumor grade and a poor prognosis in lung adenocarcinoma^23^.

FANG *et al.* reported that LINC00525 can bind to DNA upstream of the p21 promoter to form an RNA‒DNA triplex, which directs LINC00525 and its related enhancer of Zeste 2 polycomb repressive complex 2 subunit (EZH2) to the promoter region of p21, leading to an increase in the trimethylation of lysine 27 on histone H3 (H3K27me3) of the p21 promoter, which represses the transcription of p21^23^. In addition, LINC00525 regulates p21 mRNA stability by competitively binding to RBMS2 in the cytoplasm. RBMS2 binds to adenosine- and uridine-rich elements (AREs) in the p21 3'UTR to stabilize p21 mRNA. LINC00525 competitively binds to RBMS2 to promote the mutation of p21 mRNA and thereby inhibit the posttranscriptional expression of p21 (Figure 2). These findings suggest that RBMS2, in coordination with EZH2, mediates the downregulation of p21 by LINC00525 at both the transcriptional and post-transcriptional levels, ultimately promoting the progression of lung adenocarcinoma. Moreover, this model is further supported by observations from Zhou *et al.*, who reported that LINC01094 competitively binds to RBMS2 in a dose-dependent manner. This interaction disrupts the mRNA-stabilizing function of RBMS2, leading to accelerated degradation of CDKN1A mRNA—which encodes the p21 protein—and consequently contributing to enhanced malignant phenotypes^24^. However, it should be noted that current evidence for RBMS2's function remains largely confined to breast cancer and lung cancer; validation in other malignancies requires further investigation. To sum up, these results provide a compelling rationale for further exploration of RBMS2 as a promising biomarker and therapeutic target in oncology.

## 3. RBMS3

### 3.1 RBMS3 and breast cancer

Metastasis is a major cause of poor prognosis in breast cancer patients, and EMT is recognized as an important early step in cancer progression and metastasis. RBMS3 overexpression can effectively inhibit the migration and invasion of breast cancer cells and inhibit the EMT phenotype^25^. First, the results of qRT‒PCR analysis showed that the expression of RBMS3 mRNA was significantly lower in breast cancer tissues than in normal tissues. Second, the expression of RBMS3 mRNA and protein was significantly greater in breast cancer MCF-7 cells after transfection with RBMS3 than in the control group, as shown by RT‒PCR and western blot analysis. An MTT assay was used to assess the effect of RBMS3 on breast cancer cell proliferation and showed that RBMS3 overexpression significantly inhibited the proliferation of MCF-7 cells. Yang *et al.* showed that the expression of RBMS3 was significantly downregulated at both the mRNA and protein levels in human breast cancer tissues and cell lines; RBMS3 overexpression significantly inhibited the proliferation, migration and invasion of breast cancer cells *in vitro* and significantly attenuated tumor growth *in vivo*^25^.

**Figure 3:**
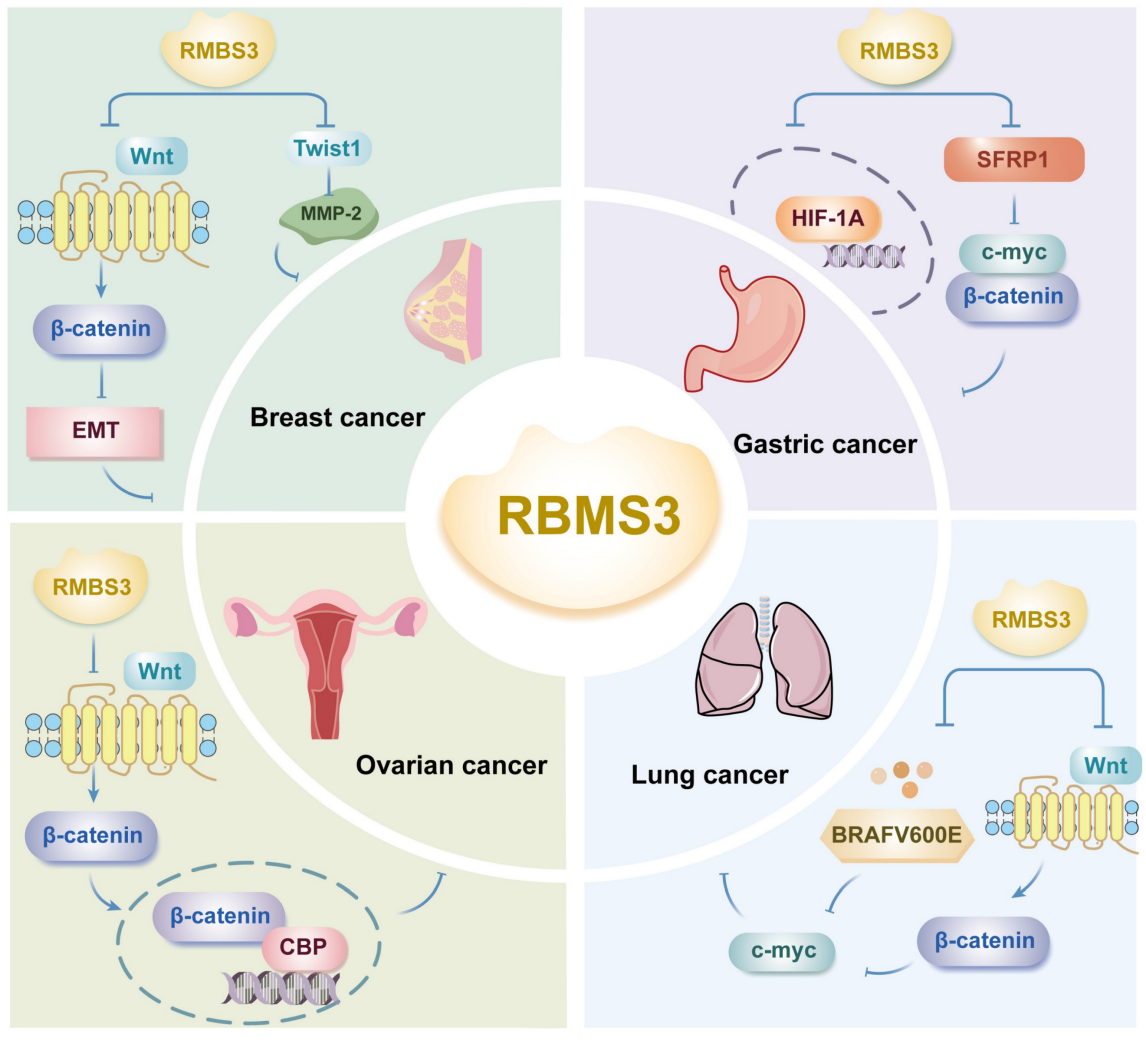
RBMS3 is involved in the development of cancer through multiple mechanisms.

Górnicki's study revealed that analysis of clinical data showed that patients who negative for RBMS3 according to immunohistochemistry (IHC) had a shorter overall survival than those positive for RMBS3; furthermore, in this study, Kaplan‒Meier analysis of 2,976 patients with breast cancer grouped by RBMS3 expression revealed that patients in the low RBMS3 expression group had significantly shorter overall survival. This finding suggested that high expression of RBMS3 at both the mRNA and protein levels is associated with longer overall survival^26^. Previous studies have confirmed that cell cycle protein D (cyclin D) is a target of Wnt signaling and that elevated levels of cyclin D is a characteristic of advanced breast cancer^27^. Yang *et al.* demonstrated that RBMS3 significantly inhibited the protein expression of β-catenin, cyclin D1 and c-Myc in MCF-7 breast cancer cells, inhibited the EMT phenotype, increased the expression of E-cadherin and decreased the expression of vimentin^25^. This study suggested that RBMS3 inhibits breast cancer cell migration and proliferation in part by inactivating the Wnt/β-catenin signaling pathway and regulating EMT and that RBMS3 may be a new potential therapeutic target for breast cancer (Figure 3).

Similarly, in triple-negative breast cancer, RBMS3 is required for the maintenance of the mesenchymal phenotype as well as invasion and migration *in vitro*^28^. In this study, Block *et al.* reported that RBMS3 interacts with the paired related homeobox 1 (PRRX1) mRNA, which regulates EMT and promotes the stabilization of PRRX1. PRRX1 is required for RBMS3-mediated EMT, and in triple-negative breast cancer cell lines, knockdown of RBMS3 through PPRX1 supplementation increased the extent of EMT in tumor cells^28^.

Furthermore, it has been shown that in breast cancer, RBMS3 binds to the Twist1 3'-UTR to negatively regulate Twist1 expression and reduce Twist1-induced matrix metalloproteinase 2 (MMP-2) expression^29^ (Figure 3). The MMP family degrades and disrupts vascular endothelial cell junctions, promotes tumor metastasis, and is responsible for the poor prognosis of breast cancer patients. Twist1-induced cell migration, invasion, and lung metastasis can be reversed by upregulation of RBMS3, suggesting that the RBMS3-mediated reduction in Twist1 expression plays a crucial role in breast cancer metastasis, revealing that the RBMS3/Twist1/MMP-2 axis regulates breast cancer invasion and metastasis via a novel mechanism^29^.

### 3.2 RBMS3 and gastric cancer

Deletion of a specific region on the short arm of chromosome 3 (3p) results in loss of function of the tumor suppressor gene (TSG), one of the most common genetic alterations in many human solid tumors, including gastric cancer^30^. RBMS3, located in this region, acts as a tumor suppressor in many cancers and mediates tumor angiogenesis^31^. Compared with corresponding normal tissues, gastric cancer tissues exhibit significantly lower expression of RBMS3 and secreted frizzled-related protein (SFRP1) at the mRNA and protein levels, respectively; moreover, there is a positive correlation between the expression levels of RBMS3 and SFRP1^32^. This study revealed that low expression of RBMS3 and SFRP1 was significantly associated with poor histologic grade and a poor prognosis in gastric cancer patients, and multivariate analysis confirmed that the coexpression status of RBMS3 and SFRP1 was an independent prognostic factor for gastric cancer patients. This study further revealed that RBMS3 and SFRP1 could exert cancer-inhibitory effects by downregulating the expression of c-Myc and β-catenin, suggesting that RBMS3 and SFRP1 may have synergistic tumor-suppressive effects^32^ (Figure 3).

On the other hand, the levels of RBMS3 and HIF1A, key regulators of angiogenesis, were detected in gastric cancer tissues and paired normal gastric tissues using quantitative PCR (qPCR) and western blotting; in gastric cancer tissues, RBMS3 was downregulated at both the mRNA and protein levels, while HIF1A was upregulated at both the mRNA and protein levels (Figure 3). Further studies revealed that HIF1A is expressed in both the cytoplasm and nucleus and has different functions in cancer progression^33^. Nuclear HIF1A is overexpressed in a variety of tumors, and its expression is often associated with a poor prognosis^34^. A study by Wu *et al.* revealed that RBMS3 was negatively correlated with nuclear HIF1A and positively correlated with cytoplasmic HIF1A in gastric cancer and that RBMS1 promoted the translocation of HIF1A from the nucleus to the cytoplasm. This study confirmed that RBMS3 and nuclear HIF1A expression were significantly associated with clinical prognosis by Kaplan‒Meier analysis and that nuclear HIF1A+ gastric cancer patients had poorer overall survival (OS) than did cytoplasmic HIF1A+ patients, suggesting that RBMS3 may regulate the cellular localization of HIF1A. In addition, univariate and multivariate Cox regression analyses supported RBMS3 expression as an independent prognostic factor in gastric cancer. The downregulation of RBMS3 and upregulation of nuclear HIF1A could lead to the development of new therapeutic molecular targets for gastric cancer^31^. After further assessing whether RBMS3 regulates GC angiogenesis, the number of full tubes formed in medium conditioned by gastric cancer cells with RBMS3 overexpression was found to be significantly lower than that in the negative control group according to the results of the endothelial cell tube formation assay, suggesting that RBMS3 may inhibit angiogenesis in gastric cancer cells *in vitro*. In summary, the combined expression of RBMS3 and nuclear HIF1A was a more reliable predictor of gastric cancer prognosis than RBMS3 expression or nuclear HIF1A expression alone. These results suggest that the combination of RBMS3 and nuclear HIF1A may be a key molecular prognostic indicator for gastric cancer patients and may provide a new approach for the treatment of gastric cancer.

### 3.3 RBMS3 and reproductive system tumors

RBMS3 deletion in epithelial ovarian cancer is correlated with overall survival and recurrence-free survival, and RBMS3 levels in ovarian cancer tissues were significantly lower than those in neighboring structures. Deletion of RBMS3 significantly enhances resistance to cisplatin (CDDP) in epithelial ovarian cancer through activation of Wnt/β-catenin/CBP signaling, whereas restoration of RBMS3 reduces chemoresistance in epithelial ovarian cancer^35^. RBMS3 was found to inhibit β-catenin/CBP signaling by directly associating with and stabilizing multiple negative regulators, including DKK3, AXIN1, BACH1, and NFAT5, thereby competitively blocking miR-126-5p-mediated inhibition of these transcripts (Figure 3). PRI-724, a second-generation CBP/β-catenin antagonist, and the combination of cisplatin (CDDP) and PRI-724 demonstrated significant therapeutic efficacy in a preclinical model of epithelial ovarian cancer with RBMS3 deletion, confirming the importance of RBMS3 deletion in chemoresistance and potentially revealing a new therapeutic strategy for epithelial ovarian cancer^36^. In multiple cancer types, Chr3p23-24.1 deletion is significantly associated with shorter recurrence-free survival and may serve as an independent prognostic factor, whereas RBMS3 (one of the genes in Chr3p23-24.1), a tumor suppressor gene whose deletion is associated with adverse effects and resistance to platinum-based treatments in epithelial ovarian cancers, may serve as a potential treatment target or prognostic biomarker for ovarian cancers or as a marker for subtyping patients based on gene expression profiles^36^.

Given the established tumor-suppressive roles of RBMS3 in ovarian cancer, its functional significance in other gynecological malignancies—particularly cervical carcinogenesis—remains a critical yet unexplored priority for future mechanistic investigations.

### 3.4 RBMS3 and lung cancer

The expression of BRAFV600E in the lung epithelium triggers the growth of benign lung tumors, which rarely progress to malignant lung adenocarcinomas in the absence of additional genetic alterations^37^. To identify genes that synergistically facilitate BRAFV600E-driven malignant progression, transposon mutagenesis was employed, revealing RBMS3 as a potential tumor suppressor. Silencing of RBMS3 promoted the growth of BRAFV600E lung-like organs and the development of malignant lung cancers with distinctive micropapillary structures in BRAFV600E and EGFR L858R genetically engineered mouse models^37^ (Figure 3). On the other hand, RBMS3 silencing enhanced signaling through the WNT/β-catenin signaling axis. Mechanistically, BRAFV600E induced WNT/β-catenin signaling to promote c-MYC expression (Figure 3). Collectively, these findings reveal a role for RBMS3 as an inhibitor of lung cancer and suggest that RBMS3 silencing may contribute to the progression of non-small cell lung cancer. Further studies are warranted to assess the clinical relevance of RBMS3 in larger lung cancer cohorts and to explore its potential as a biomarker or therapeutic target.

## 4. Outlook

The study of the RBMS family is highly important for exploring the development of malignant tumors, and the elaboration of the pathways and molecular mechanisms of RNA-binding proteins associated with cancer progression is a hot topic of current research. In this paper, we reviewed in detail the expression of the RBMS family in different malignant tumors and the progress of related research on the mechanism of action. This study provides relevant reference information for therapeutic approaches based on the RBMS as a target and is expected to promote the development and application of the RBMS family in the field of malignant tumor diagnosis and treatment. The functional heterogeneity of the RBMS family has been demonstrated in several studies, and although the RBMS family plays an inhibitory role in most malignant tumors, RBMS can promote metastasis and recurrence in a few tumors. Additional in-depth studies are needed to reveal the functional mechanisms of different members of the RBMS family in the exploration of the value of RBMS proteins as clinical diagnostic and prognostic markers and therapeutic targets.

In our perspective, the RBMS family represents a promising yet underexplored class of RNA-binding proteins with significant potential as therapeutic targets. Future studies should focus on several key aspects: First, the development of small molecule inhibitors or activators that specifically target individual RBMS proteins (e.g., RBMS1 inhibitors for lung cancer or RBMS3 activators for ovarian cancer) could provide novel therapeutic avenues. Second, the striking context-dependent roles of RBMS proteins across different cancer types (e.g., RBMS1 acting as a tumor suppressor in colorectal cancer but as an oncogene in lung cancer) necessitate more comprehensive studies to elucidate the underlying mechanisms, potentially through single-cell sequencing and spatial transcriptomics technologies. Finally, the exploration of RBMS proteins as biomarkers for cancer diagnosis and prognosis should be expanded to larger clinical cohorts, and their potential utility in liquid biopsies deserves particular attention. We believe that addressing these challenges will not only deepen our understanding of RBMS proteins in carcinogenesis but also accelerate their translation into clinical applications.

## Figures and Tables

**Table 1:** RBP Targets and Dysregulation Associated with Cancer

RBP	targets	Cancer type	Mechanism/signaling pathway/conclusion	Biological functions	Reference
RBMS1	GPX4	Hepatocellular carcinoma	Regulates GPX4 mRNA stability via circIDE/miR-19b-3p axis, promoting ferroptosis	Promotes ferroptosis	3
	IL-6	Gastric cancer	Activates IL-6/JAK2/STAT3 autocrine signaling pathway	Promotes migration and proliferation	4
	KCNQ1OT1/miR-378a-3p	Gastric cancer	Activated by KCNQ1OT1 ceRNA sponging miR-378a-3p; positively correlated with immune cell infiltration	Modulates immune microenvironment	6
	AKAP12; SDCBP	Colon cancer	Stabilizes AKAP12/SDCBP mRNAs to suppress metastasis	Suppresses metastasis	9,10
	miR-4442	Colon cancer	Regulates EMT through miR-4442-mediated targeting	Inhibits EMT	11
	SLC7A11	Lung cancer	Promotes SLC7A11 translation via eIF3d interaction, inhibiting ferroptosis	Inhibits ferroptosis	12,13
	circEXOC6B	Prostate cancer	circEXOC6B binds to RBMS1 and HuR, and regulates the stability of AKAP12	Suppresses metastasis	15,16
RBMS2	BMF	Breast cancer	Stabilizes BMF mRNA to enhance doxorubicin-induced apoptosis	Promotes apoptosis	17
	P21	Breast cancer	Stabilizes P21 mRNA to inhibit proliferation via p53 pathway	Inhibits proliferation	18
	P21	Lung cancer	LINC00525 promotes p21 mRNA decay by competitively binding to RBMS2	Promotes tumorigenesis	20,23
RBMS3	β-catenin; Cyclin D1	Breast cancer	Inactivates Wnt/β-catenin signaling pathway	Inhibits proliferation	25,26,27
	PRRX1	Breast cancer	Stabilizes PRRX1 mRNA to maintain mesenchymal phenotype	Promotes EMT	28
	Twist1/MMP-2	Breast cancer	Binds Twist1 3'UTR to suppress MMP-2 expression	Inhibits invasion and metastasis	29
	SFRP1; c-Myc	Gastric cancer	Downregulates c-Myc/β-catenin with SFRP1	Suppresses tumor progression	31,32
	HIF1A	Gastric cancer	Promotes HIF1A cytoplasmic translocation to inhibit angiogenesis	Inhibits angiogenesis	33,34
	Wnt/β-catenin/CBP signaling	Ovarian cancer	Inhibits β-catenin/CBP signaling by stabilizing negative regulators via miR-126-5p axis, sensitizing to cisplatin	Reverses chemoresistance	35,36
	WNT/β-catenin	Lung cancer	Silencing enhances BRAFV600E-driven tumor progression via WNT/β-catenin/c-MYC axis	Suppresses tumor progression	37
